# Storm-event-transport of urban-use pesticides to streams likely impairs invertebrate assemblages

**DOI:** 10.1007/s10661-016-5215-5

**Published:** 2016-05-12

**Authors:** Kurt D. Carpenter, Kathryn M. Kuivila, Michelle L. Hladik, Tana Haluska, Michael B. Cole

**Affiliations:** Oregon Water Science Center, 2130 SW 5th Avenue, Portland, OR 97201 USA; California Water Science Center, 6000 J Street, Placer Hall, Sacramento, CA 95819 USA; Cole Ecological, Inc., 15 Bank Row, Suite B, Greenfield, MA 01301 USA

**Keywords:** Urban streams, Pesticides, Source, Transport, Bifenthrin, Fipronil, Benthic invertebrates

## Abstract

Insecticide use in urban areas results in the detection of these compounds in streams following stormwater runoff at concentrations likely to cause toxicity for stream invertebrates. In this 2013 study, stormwater runoff and streambed sediments were analyzed for 91 pesticides dissolved in water and 118 pesticides on sediment. Detections included 33 pesticides, including insecticides, fungicides, herbicides, degradates, and a synergist. Patterns in pesticide occurrence reveal transport of dissolved and sediment-bound pesticides, including pyrethroids, from upland areas through stormwater outfalls to receiving streams. Nearly all streams contained at least one insecticide at levels exceeding an aquatic-life benchmark, most often for bifenthrin and (or) fipronil. Multiple U.S. EPA benchmark or criterion exceedances occurred in 40 % of urban streams sampled. Bed sediment concentrations of bifenthrin were highly correlated (*p* < 0.001) with benthic invertebrate assemblages. Non-insects and tolerant invertebrates such as amphipods, flatworms, nematodes, and oligochaetes dominated streams with relatively high concentrations of bifenthrin in bed sediments, whereas insects, sensitive invertebrates, and mayflies were much more abundant at sites with no or low bifenthrin concentrations. The abundance of sensitive invertebrates, % EPT, and select mayfly taxa were strongly negatively correlated with organic-carbon normalized bifenthrin concentrations in streambed sediments. Our findings from western Clackamas County, Oregon (USA), expand upon previous research demonstrating the transport of pesticides from urban landscapes and linking impaired benthic invertebrate assemblages in urban streams with exposure to pyrethroid insecticides.

**Electronic supplementary material:**

The online version of this article (doi:10.1007/s10661-016-5215-5) contains supplementary material, which is available to authorized users.

## Introduction

Reductions in the abundance or diversity of aquatic insects can have important consequences for aquatic ecosystems, particularly for young salmon and steelhead that consume aquatic invertebrates (National Marine Fisheries Service 2012), but also birds and bats that feed on the adult insects that hatch from streams (Baxter et al. 2005). Many urban streams in the greater Portland, Oregon, metropolitan area once supported native salmonid populations, but populations have since declined substantially (Oregon Department of Fish and Wildlife 2010). The low numbers reflect combined habitat and water quality impairment and changes in the base of the food web, including reduced numbers of insects and dominance by less desirable organisms such as oligochaetes, nematodes, and flatworms (Waite et al. 2008). Severely disturbed aquatic invertebrate populations have been found in many of the urban streams in and around Portland (Mulvey et al. 2009; Waite et al. 2008) and in Clackamas County (Cole 2014; Lemke et al. 2013; Dewberry et al. 1999). Although the specific causes for their impaired condition have not been fully evaluated, exposure to insecticides could play a role.

Frequent detection of insecticides at high concentrations in urban streams nationally (Stone et al. 2014) suggests that exposure to these compounds is another stressor likely to impact aquatic invertebrates. Previous studies of urban and rural/agricultural streams in the nearby Clackamas River Basin (Carpenter 2004; Carpenter et al. 2008) found numerous pesticides in stormwater runoff (11 compounds per sample, on average), with several pesticides exceeding U.S. EPA chronic benchmarks for invertebrates (U.S. Environmental Protection Agency 2014), oftentimes for multiple insecticides simultaneously. Since then, new types of insecticides have increased in use, particularly pyrethroids (bifenthrin in particular), and fipronil, a phenyl pyrazole insecticide. Now, residues of these insecticides are showing up in some of California’s urban streams at levels of concern (Weston et al. 2014; Ensminger et al. 2013).

While fipronil’s high water solubility allows transport of the dissolved compound from the landscape to receiving stream, bifenthrin and other hydrophobic pyrethroids demonstrate a strong tendency to associate with fine sediment and organic matter (Gan et al. 2005a). Pyrethroid compounds transported in stormwater runoff may settle out into streambed sediments (Kuivila et al. 2012) and cause harm to benthic invertebrates (Nowell et al. 2013; Moran et al. 2012). While these new pesticides tend to be less toxic to mammals (U.S. EPA 2011), they are very toxic to aquatic organisms (Siegfried 1993 and references cited therein). Their high frequency of detection in streams highlights the importance of understanding the sources, transport mechanisms, and factors affecting toxicity, including properties of the sediments (organic carbon and (or) sand content) and water temperature (Holmes et al. 2008; Weston et al. 2011), so that strategies can be developed to minimize potential impacts on aquatic life.

### Study background and objectives

This study was conducted, in part, to satisfy the Oregon Department of Environmental Quality (ODEQ’s) requirement for the new National Pollutant Discharge Elimination System (NPDES) Municipal Separate Storm Sewer System (MS4) permits issued to the Clackamas County Co-permittees in 2012. Our scientific objectives were to evaluate the sources, transport, and fate of current-use insecticides in these streams and assess possible adverse effects on benthic invertebrates using measured pesticide concentrations and existing invertebrate data (Lemke et al. 2013; Cole 2014) collected as part of the MS4 permit.

## Methods

### Site selection and data collection

Sites were selected to represent the range of urban development, with priority given to sites where invertebrate monitoring was completed. Stormwater and sediment samples were collected from 12 urban streams, 5 paired stormwater outfalls, and 3 streams draining mixed basins including some agricultural land (Table 1, SI 1, and SI 2). Although these 3 were included in the study for comparison, one site (Rock Creek) was included along with the other predominantly urban streams in some of the analyses given the high-density development in the watershed and the availability of comparable invertebrate community data.

**Table 1:** List of stormwater outfall and stream sampling sites, basin characteristics, select pesticide concentrations in stormwater runoff and streambed sediments, and benthic invertebrate assemblage disturbance class

Map no.	Site	% urban	% impervious area	Storm runoff SSC (mg/L)	Stormwater runoff concentrations^a^ for pesticides exceeding aquatic-life benchmarks or criteria				Streambed sediment concentrations (μg/kg)		
					Bifenthrin	Fipronil	Malathion	DDE + DDD	Total pyrethroids	Total DDT degradates	Invertebrate assemblage disturbance class^b^
**Stormwater outfalls and receiving streams**											
1	Outfall to Tanner Creek	97	42	68	120*	30*	<	<	1700	–	–
2	Tanner Creek	77	40	136	97*	127**	<	<	34	<	Severe
3	Outfall to Lost Dog Creek	65	27	85	31*	59*	<	<	246	–	–
4	Lost Dog Creek	52	21	102	24*	16*	<	1.1***	73.9	2.3	Severe
5	Outfall to Rose Creek/Sieben Creek	100	78	104	32*	<	<	<	304	–	–
6	Sieben Creek	62	35	545	39*	10	<	9.2***	1.7	0.9	Moderate
7	Outfall to Kellogg Creek	94	43	6.2	<	6.1	<	<	240	1.7	–
8	Kellogg Creek	76	42	76	21*	10.5	<	<	3.1	1.2	Moderate
9	Outfall to detention pond, Wilsonville^c^	65	66	152	29*	<	<	<	190	–	–
**Other predominantly urban streams**											
11	Ball Creek	83	35	89	21*	19*	<	<	4.1	<	Severe
10	Boeckman Creek (lower)	39	20	32	<	<	<	<	1.2	<	Severe
13	Carli Creek	96	74	105	23*	<	<	<	8.8	<	Severe
14	Coffee Creek	82	39	200	23*	6.7	<	<	1	0.7	Slight
16	Minthorn Spring Creek	92	47	339	24*	6.4	<	<	3.5	4	Severe
18	Singer Creek	83	37	247	<	<	457**	<	30.4	1.1	Slight-moderate
19	Singer Creek tributary	77	34	31	<	20*	<	<	<	<	nd
20	Trilium Creek	68	29	162	24*	12*	<	<	30.3	1.1	Moderate-severe
**Streams draining some agricultural land**											
12	Boeckman Creek (upper)	29	15	1770	31*	<	<	<	<	<	nd
15	Deep Creek	8	4	138	22*	<	<	2.7***	<	0.8	nd
17	Rock Creek	26	12	34	<	12*	<	<	<	0.7	Moderate-severe

Map is shown in SI 1. Percent urban and impervious areas from National Land Cover Data (NLCD; Fry et al. 2011; impervious area data updated in 2014). Urban area includes NLCD classes 22, 23, and 23 (low-, medium-, and high-intensity urban). Pesticide exceedances indicated with asterisk(s) according to: * Exceeds the U.S. EPA Office of Pesticide Programs 21-day chronic benchmark for invertebrates of 1.3 ng/L bifenthrin or 11 ng/L fipronil; ** Exceeds the U.S. EPA Office of Pesticide Programs acute benchmark for invertebrates of 110 ng/L fipronil or 300 ng/L malathion; *** Exceeds water-quality criterion of 1 ng/L for total DDT plus degradates established by the Clean Water Act for the protection of aquatic life

*SSC* suspended sediment concentration, *mg/L* milligrams per liter, *nd* no data; <, less than MDL (see Table SI 4); –, not applicable

^a^Stormwater runoff values are whole-water concentrations (sum of the dissolved and suspended fractions) in ng/L

^b^Based on the Oregon Department of Environmental Quality invertebrate metric scores presented in Lemke et al. (2013) and Cole (2014)

^c^This outfall does not discharge to Boeckman Creek

Pesticide samples were collected August–September 2013, starting with fine-grained streambed sediment sampling at 14 streams during the late summer low-flow period. Stream-deposited sediments were targeted for sampling and care was taken to avoid sediments derived from adjacent eroding banks. Sampling and processing equipment were cleaned with Liquinox™ soap, rinsed with distilled/deionized water, methanol, and certified organic-free water. Streambed sediment subsamples were collected with a stainless-steel spoon from the top 2 cm of sediment from 10 to 15 locations at each site and composited. The sediment slurry was homogenized and sieved (2-mm stainless steel) into clean 250-mL glass jars.

Samples of stormwater runoff were collected on September 5–6, 2013, following about an inch of rain. Whole-water samples were collected by directly filling 1-L baked amber-glass bottles using a width-integrated method for streams and point-grab samples for stormwater outfalls. Based on continuous monitoring data from nearby Fanno Creek (SI 3), this storm was considered a “first flush” event for the season, producing a characteristic peak in turbidity that subsided with additional rainfall, presumably through dilution and decreased mobilization of sediments.

**Fig. 1:**
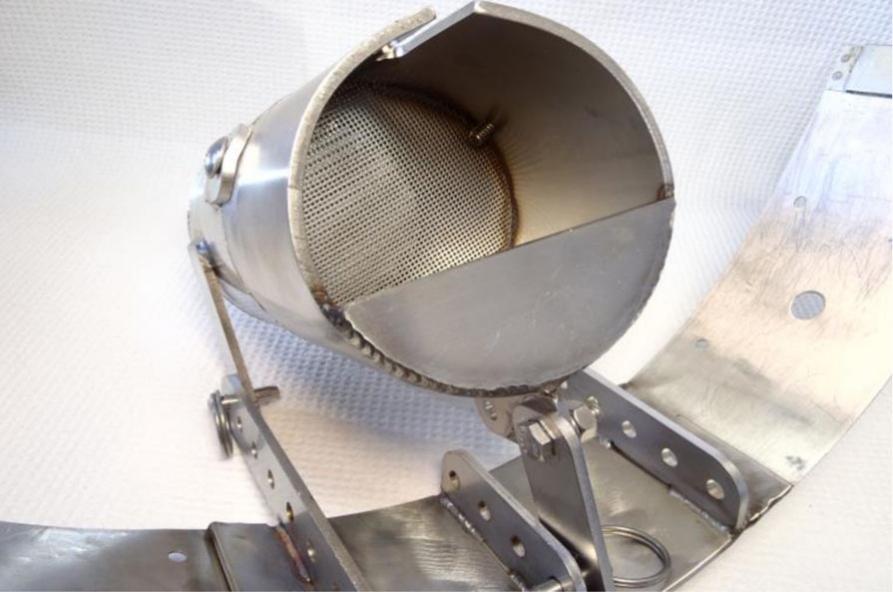
Screened Inline Flow-Through (SIFT) sediment trap

Prior to the storm, stainless steel Screened Inline Flow-Through (SIFT©) sediment traps designed by the City of Portland (Fig. 1) were deployed in 3 outfalls: Rose Creek/Sieben Creek outfall, Kellogg Creek outfall, and the outfall in Wilsonville (SI 1), in a pilot effort to monitor for pesticides on sediments transported in stormwater from the “pipe-shed.” The samplers collected time-integrated samples of sediments >226 μm (Randy Belston, City of Portland, written communication, 2013). Samplers were deployed on July 17 and sediments retrieved from all three outfalls on September 13, a week after the September 5–6 storm. Due to low volumes of sediment retrieved from the Rose Creek/Sieben Creek and Kellogg Creek outfalls, SIFT sediment traps were redeployed for another 38 days until October 21, when a second set of post-storm samples was retrieved.

Invertebrate assemblage data (and community metrics) were assessed alongside pesticide concentrations to examine for possible effects. Benthic macroinvertebrate samples were collected at all sites in either 2011 or 2013 using Oregon DEQ’s protocol for wadeable rivers and streams (ODEQ 2009). Specific details are presented in Lemke et al. (2013) and Cole (2014). Briefly, targeted riffle samples (8-kick composites) were collected using a 500-μm mesh D-frame net. Samples were sorted to remove a 500-organism subsample. Identifications were performed by Michael Cole and Ann Gregoire, Cole Environmental, Inc. Most aquatic insects, including Ephemeroptera, Plecoptera, and Tricoptera, and other arthropods were identified to genus or species. Mollusks were identified to family or genus, oligochaetes were identified to class, and chironomids were identified to subfamily/tribe.

### Pesticide analyses and quality assurance

Analyses included 91 pesticides dissolved in water and 118 compounds on sediment (SI 4). Pesticide samples were analyzed at the USGS Organic Chemistry Laboratory in Sacramento, CA, using methods for water (Hladik et al. 2008), suspended sediment (Hladik et al. 2009), and streambed sediment (Hladik and McWayne 2012). Stormwater samples were processed through 0.7-μm glass-fiber filters in the laboratory, with dissolved and suspended fractions analyzed separately using gas chromatography–mass spectroscopy (GC/MS). Method detection limits were 0.9 to 10.5 ng/L for water and 0.5 to 3.1 μg/kg for sediment (SI 4).

Percent organic carbon concentrations were determined for streambed and SIFT sediments at the USGS Organic Chemistry Laboratory using a PerkinElmer CHNS/O analyzer (Perkin Elmer Corporation, Waltham, MA). Before analysis, sediments were dried to a constant weight at 110 °C for 3 h. Sediments were combusted at 925 °C in silver boats after being exposed to concentrated hydrochloric acid fumes in a desiccator for 24 h to remove inorganic carbon. Acetanilide was used for instrument calibration for carbon.

Quality assurance samples included one field blank, three replicates, four matrix spikes, plus two surrogate matrix spikes for each sample analyzed. There were no QA issues identified; the blank sample was clean (SI 5), and relative percent differences (RPD) between replicate samples were < 20 %, with an average RPD of 8.5 % (SI 6). Percent recoveries were 70–121 % (SI 7). Percent recoveries for surrogate spikes for atrazine (84–112 %) and permethrin (76–107 %) averaged 100 and 95 %, respectively (SI 8).

### Site basin characteristics

Stream and outfall basin areas were delineated for each site using light imaging detection and ranging (LiDAR), 10-m digital elevation maps, or hand delineation with guidance from staff with local jurisdictions. GIS was used to derive basin statistics from USGS Streamstats (U.S. Geological Survey 2012), the 2006 National Land Cover Data (NLCD, Fry et al. 2011), 2013 census data, and SSERGO soils data (Natural Resources Conservation Service 2014). Percent impervious area was derived using the 2001, 2006, and 2011 NLCD (posted October 2014).

Stormwater outfalls drained the most densely urbanized areas with the exception of the outfall to Lost Dog Creek, which contains some “unpiped” areas (see footnote in Table 2). These basins were 93–100 % urban, with substantial impervious areas (Table 1). The outfall to Rose Creek/Sieben Creek drains a basin that is 100 % commercial/retail, whereas other outfalls drained basins with higher amounts of residential property. Streams drain mixed basins containing low-, medium-, and high-density development, with some industrial land (SI 2). Three sites (upper Boeckman, Rock, and Deep Creeks) also contained some agricultural land (row crops and nurseries) (SI 2).

**Table 2:** Pesticide concentrations in stormwater outfall discharge and SIFT sediments

Pesticide (type)	Detection frequency (%)	Outfall to Lost Dog Creek^a^	Outfall to Tanner Creek	Outfal to Rose Creek/Sieben Creek		Outfall to Kellogg Creek		Outfall to detention pond, Wilsonville
**Stormwater discharge**								
Bifenthrin (I)	80	37 (240)	120 (1697)	32 (304)	–	<	–	29 (190)
Fipronil (I)	60	59	30	<	–	6.1	–	<
Metolachlor (H)	60	<	13	<	–	6	–	72
Carbaryl (I)	40	50	13	<	–	<	–	<
Fipronil sulfide (D)	40	9.4	3.5	<	–	<	–	<
Iprodione (F)	40	15 (145)	<	<	–	<	–	<
Kresoxim-methyl (F)	40	6 (58)	<	<	–	<	–	12 (76)
Zoxamide (F)	40	9 (91)	<	<	–	<	–	28 (187)
Boscalid (F)	20	8.6	<	<	–	<	–	<
Esfenvalerate (I)	20	6.2	<	<	–	<	–	<
Fenbuconazole (F)	20	7.2	<	<	–	<	–	<
Fipronil desulfinyl (D)	20	<	<	<	–	10.5	--	<
Flusilazole (F)	20	6.3	<	<	–	<	–	<
Piperonyl butoxide (S)	20	18	<	<	–	<	–	<
**SIFT sediments**				Sample 1	Sample 2	Sample 1	Sample 2	Sample 1
Bifenthrin (I)	100	–	–	24	436	12.1	11.5	179
Pendimethalin (H)	100	–	–	20	849	4.9	6.2	380
Trifluralin (H)	80	–	–	<	40	21	1.6	49
Dithiopyr (H)	60	–	–	12	244	<	<	176
Prodiamine (H)	40	–	–	<	39	<	<	92
DDE (D)	20	–	–	<	<	1.7	<	<
Pentachloroanisole (D)	20	–	–	1.2	<	<	<	<
Oxyfluorfen (H)	20	–	–	<	<	12.5	<	<
Methoprene (I)	20	–	–	<	<	<	25	<

Whole-water pesticide concentrations in stormwater outfall discharge in ng/L; suspended sediment concentrations in stormwater outfall discharge in μg/kg (shown in parens).

SIFT sediment pesticide concentrations in μg/kg

Pesticide types: *F* fungicide, *H* herbicide, *I* insecticide, *S* synergist, *D* pesticide degradate. <, less than MDL (see Table SI 4); –, no data

^a^Although this sampling site is classified as an outfall, about 27 % (40 acres) of the upstream watershed is “unpiped” area drained by surface channels

Several sites (Tanner, Lost Dog, Trillium, and Coffee Creeks) drain steep, highly dissected hill slopes with high drainage density (stream miles per mi^2^) that rapidly transport stormwater runoff. These include the bluffs in Lake Oswego and West Linn, which have residential landscaping that might be subject to pesticide applications. This combination of factors may present the right conditions for pesticides to mobilize to streams during storm runoff.

### Data reduction and statistical analysis

Pesticide concentrations in stormwater runoff—whole-water sums of dissolved and suspended fractions—were evaluated against U.S. EPA Office of Pesticide Programs (OPP) chronic and acute benchmarks for invertebrates and, for DDT degradates, water quality criteria set forth in the Clean Water Act for the protection of aquatic life. Benchmark quotient (BQ) values were calculated for each detection in water: BQ = sample concentration/benchmark or criteria. Based on this screening process, bifenthrin, fipronil, malathion, and the sum of DDT degradates (compounds with BQ >1) were identified as having the greatest potential for affecting invertebrate assemblages in these streams. Pesticide concentrations in streambed sediments were compared against benchmarks proposed by Nowell et al. 2016. Analyses were performed using both raw and organic-carbon-normalized bifenthrin concentrations in bed sediment to examine for potential effects on invertebrates.

Pesticide variables (concentrations, OC-normalized concentrations, and pesticide class sums) were collated with the site basin statistics and data on benthic invertebrates, water quality (temperature, dissolved oxygen, and specific conductance), and habitat conditions (riparian buffer width, bank stability, large wood, and substrate size and embeddedness) reported in Lemke et al. (2013) and Cole (2014). Most sites were sampled for benthic invertebrates a few weeks prior to the storm sampling, but four streams included in the analysis—Carli, Sieben, Rock, and Kellogg Creeks—were sampled in September 2011 (Lemke et al. 2013).

Non-metric multidimensional scaling (NMDS) ordinations, Bio-Env Stepwise (BEST) analyses, and spearman rank correlations were performed using the multivariate statistical package PRIMER (Clarke and Gorley 2006). Analyses were conducted separately on (1) all sites and (2) the predominantly urban streams without 2 of the 3 agriculturally affected sites (SI 2). As described above, Rock Creek was included as an urban affected stream in the analyses involving the invertebrates.

NMDS ordination was used to portray the pattern in the invertebrate species assemblage data based on Bray-Curtis similarity using square-root-transformed abundance data. Invertebrate assemblage traits and metrics were then examined for correlation with NMDS axes scores to understand the underlying patterns among sites. Associations between the invertebrate species composition and pesticide/environmental data matrix were examined using BEST to identify possible factor(s) that may relate to or explain the distribution of samples in the ordination. Overlay bubble plots were made to visualize relationships between the invertebrate assemblages and pesticide concentrations and other environmental variables.

## Results and discussion

Overall, 33 pesticide compounds, including 9 insecticides, were detected. Pesticides were detected at all sites in one or more sample types/phases, with up to 12 pesticides detected per site. Four compounds—bifenthrin, fipronil, a DDT degradate, and metolachlor—composed half of all detections (Tables 2 and 3).

**Table 3:** Pesticide concentrations in streams during stormwater runoff and in streambed sediments

Pesticide (type)	Predominantly urban streams													Streams draining some agricultural land		
	Detection frequency (%)	Tanner Cr.	Lost Dog Cr.	Sieben Cr.	Kellogg Cr.	Ball Cr.	Boeckman Creek (lower)	Carli Cr.	Coffee Cr.	Minthorne Spg. Cr.	Singer Cr.	Singer Cr. tributary	Trillium Cr.	Boeckman Creek (upper)	Deep Cr.	Rock Cr.
**Stormwater runoff** ^a^																
Bifenthrin (I)	73	97 (672)	24 (284)	39 (72)	21 (277)	21 (235)	<	23 (218)	23 (114)	24 (70)	<	<	24 (146)	31 (17)	22 (159)	<
Fipronil (I)	67	127	16	10	11	19	<	<	6.7	6.4	<	20	12	<	<	12
Metolachlor (H)	67	9.1	<	7.2	7.2	10.2	11	<	14	5.4	45	<	7.8	22	<	<
Carbaryl (I)	27	135	15	66	<	<	<	<	<	<	<	<	9	<	<	<
DDE (D)	20	<	1 (13)	4 (7)	<	<	<	<	<	<	<	<	<	<	3 (20)	<
Pendimethalin (H)	20	<	<	25 (46)	<	<	<	<	<	<	<	<	<	36 (20)	39 (279)	<
Propiconazole (F)	20	<	<	<	57	<	<	215	<	<	<	<	<	<	83	<
Iprodione (F)	13	<	<	21 (39)	<	<	<	<	<	<	<	<	<	<	26.9	<
Zoxamide (F)	13	<	<	14 (25)	<	<	<	<	<	<	<	<	<	<	<	22
Azoxystrobin (F)	7	<	<	<	<	<	<	<	<	<	<	<	<	<	63	<
DCPA (H)	7	<	<	<	<	<	<	10	<	<	<	<	<	<	<	<
Kresoxim-methyl (F)	7	<	<	8 (15)	<	<	<	<	<	<	<	<	<	<	<	<
Malathion (I)	7	<	<	<	<	<	<	<	<	<	457	<	<	<	<	<
Malathion-oxon (D)	7	<	<	<	<	<	<	<	<	<	47	<	<	<	<	<
DDD (D)	7	<	<	5 (9)	<	<	<	<	<	<	<	<	<	<	<	<
Simazine (H)	7	<	<	<	<	<	<	<	<	<	<	<	108	<	<	<
**Streambed sediments**																
Bifenthrin (I)	71	34	27	1.7	3.1	4.1	1.2	8.8	1	3.5	<	–	2.5	<	<	<
DDE (D)	64	<	2.3	0.9	1.2	<	<	<	0.7	2.7	1.1	–	1.1	<	0.8	0.7
Trifluralin (H)	29	6.4	3.1	1.5	<	2	<	<	<	<	<	–	<	<	<	<
Dithiopyr (H)	29	2.8	1.4	<	<	<	<	2.2	<	<	<	–	1	<	<	<
Metalaxyl (F)	21	<	<	<	<	<	21	<	<	20	392	–	<	<	<	<
Cypermethrin (I)	14	<	<	<	<	<	<	<	<	<	30	–	28	<	<	<
Pentachloroanisole (D)	14	<	2.8	<	<	<	<	3.7	<	<	<	–	<	<	<	<
Cyfluthrin (I)	7	<	47	<	<	<	<	<	<	<	<	–	<	<	<	<
Fenpyroximate (I)	7	15.1	<	<	<	<	<	<	<	<	<	–	<	<	<	<
Pendimethalin (H)	7	<	<	<	<	<	<	32	<	<	<	–	<	<	<	<
Prodiamine (H)	7	<	<	<	<	<	<	8.4	<	<	<	–	<	<	<	<
Oxyfluorfen (H)	7	<	<	<	68	<	<	<	<	<	<	–	<	<	<	<
DDD (D)	7	<	<	<	<	<	<	<	<	1.3	<	–	<	<	<	<

Stormwater runoff pesticide concentrations in ng/L (suspended sediment concentrations in μg/kg in parens). Streambed sediment pesticide concentrations in μg/kg

Pesticide types: *F* fungicide, *H* herbicide, *I* insecticide, *D* degradate; <, less than MDL (see Table SI 4); –, no data

^a^Stormwater runoff values are whole-water concentrations (sum of the dissolved and suspended fractions)

Twenty samples of stormwater runoff from outfalls and streams resulted in the detection of 18 pesticides, mostly fungicides and insecticides (Tables 2 and 3). The most frequently detected pesticides were two insecticides, bifenthrin and fipronil, which occurred in 80 and 60 % of samples from stormwater outfalls, respectively, and 73 and 67 % of samples from streams (Tables 2 and 3). The highest concentration of bifenthrin (120 ng/L) occurred in the outfall to Tanner Creek, with the next highest concentration in Tanner Creek. Tanner Creek also contained the highest concentration of fipronil (127 ng/L), with the next highest concentration occurring in the outfall to Lost Dog Creek (Table 1). These outfall-stream systems drain relatively high-elevation neighborhoods with large single-family houses, often with large lawns and manicured landscaping; the latter stream site drains a mixed basin containing residential and commercial land, and a golf course (SI 2). These watersheds are also relatively steep, making for rapid transport of runoff during storms that is often highly turbid.

The frequent detection of bifenthrin and fipronil is consistent with their use in urban environments (SI 9) and their relatively long half-lives (many months to over 1 year; SI 10). Although bifenthrin and fipronil are less toxic to mammals (U.S. EPA 2011), they are much more toxic to cold-blooded aquatic invertebrates, which may have consequences for stream life, including the small organisms that fish and other creatures feed upon.

Changes in pesticide use over the past decade have likely resulted in bifenthrin and fipronil replacing organophosphate insecticides such as diazinon, which was banned for urban use in 2005 (Ryberg et al. 2010; U.S. Environmental Protection Agency 2012). While diazinon was not detected in our current study, it was found in 25 % of samples during a previous Clackamas River Basin study conducted in 2000–2005 (Carpenter et al. 2008), including Carli, Sieben, and Rock Creeks sampled for the current study.

The most frequently detected herbicide was metolachlor, which occurred in about two thirds of stormwater samples collected from the outfalls and streams. In Oregon, metolachlor is only used by licensed applicators, for control of grasses and small-seeded broadleaf weeds. Its high frequency of detection is consistent with a previous study in the Clackamas River basin (Carpenter et al. 2008) that found metolachlor in nearly half of over 100 samples, including detection in raw and finished (treated) drinking water.

### Partitioning of pesticides in stormwater runoff

**Fig. 2:**
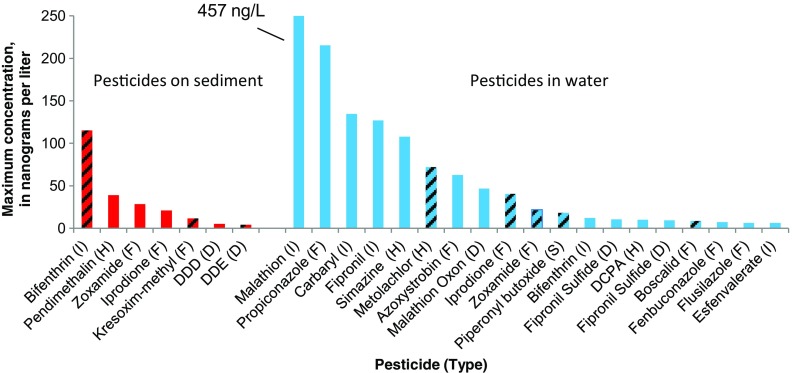
Maximum concentrations of pesticides in stormwater runoff. Sampling included 15 streams and 5 outfall sites, each sampled once. Bar cross hatches indicate pesticides with maximum concentrations in stormwater outfalls. Pesticide types: *F*, fungicide; *H*, herbicide; *I,* insecticide; *S*, synergist; *D*, degradate

Samples of stormwater runoff were filtered to allow analyses of dissolved and suspended (filter retained) fractions (SI 11 and SI 12). Seven pesticides partitioned onto suspended sediment, with one or more insecticides occurring on sediment in nearly three quarters of samples (Tables 2 and 3). Nearly three times as many dissolved pesticides were detected and at higher concentrations (Fig. 2), despite the high concentrations of total suspended sediments (Table 1). Differences in method detection limits for dissolved versus sediment (SI 4), however, may also affect detection frequencies.

**Fig. 3:**
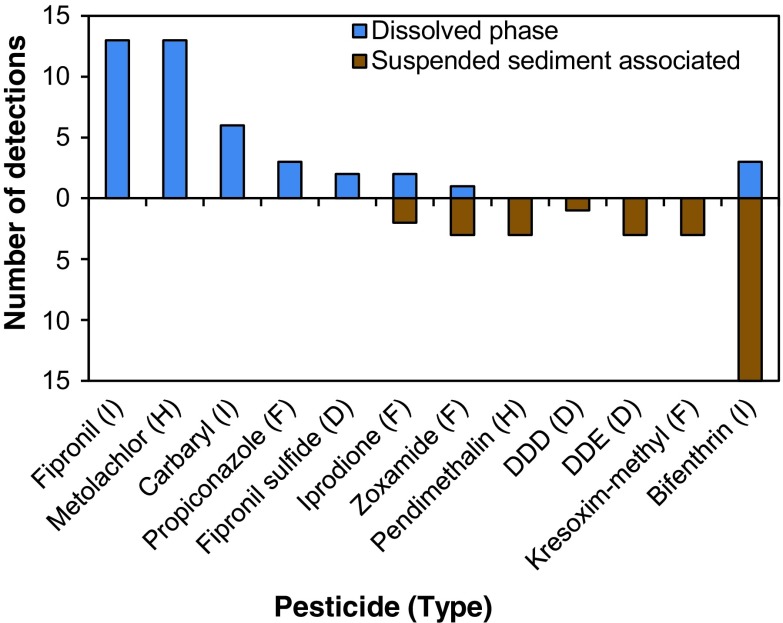
Comparison of dissolved and suspended sediment associated pesticides in stormwater. Includes compounds detected in >1 sample (*n* = 20 samples). Pesticide types: *F*, fungicide; *H*, herbicide; *I*, insecticide; *D*, pesticide degradate

Fipronil, metolachlor, carbaryl, and propiconazole occurred exclusively in the dissolved phase, whereas bifenthrin, kresoxim-methyl, DDT degradates, pendimethalin, and zoxamide had their greatest frequency of detection on suspended sediments (Fig. 3). The partitioning of these pesticides into dissolved and sediment phases is consistent with their water solubilities and Koc values (SI 10), and points to the importance of both fractions in transporting pesticides during storms.

### Transport of pesticides from outfalls to streams

**Fig. 4:**
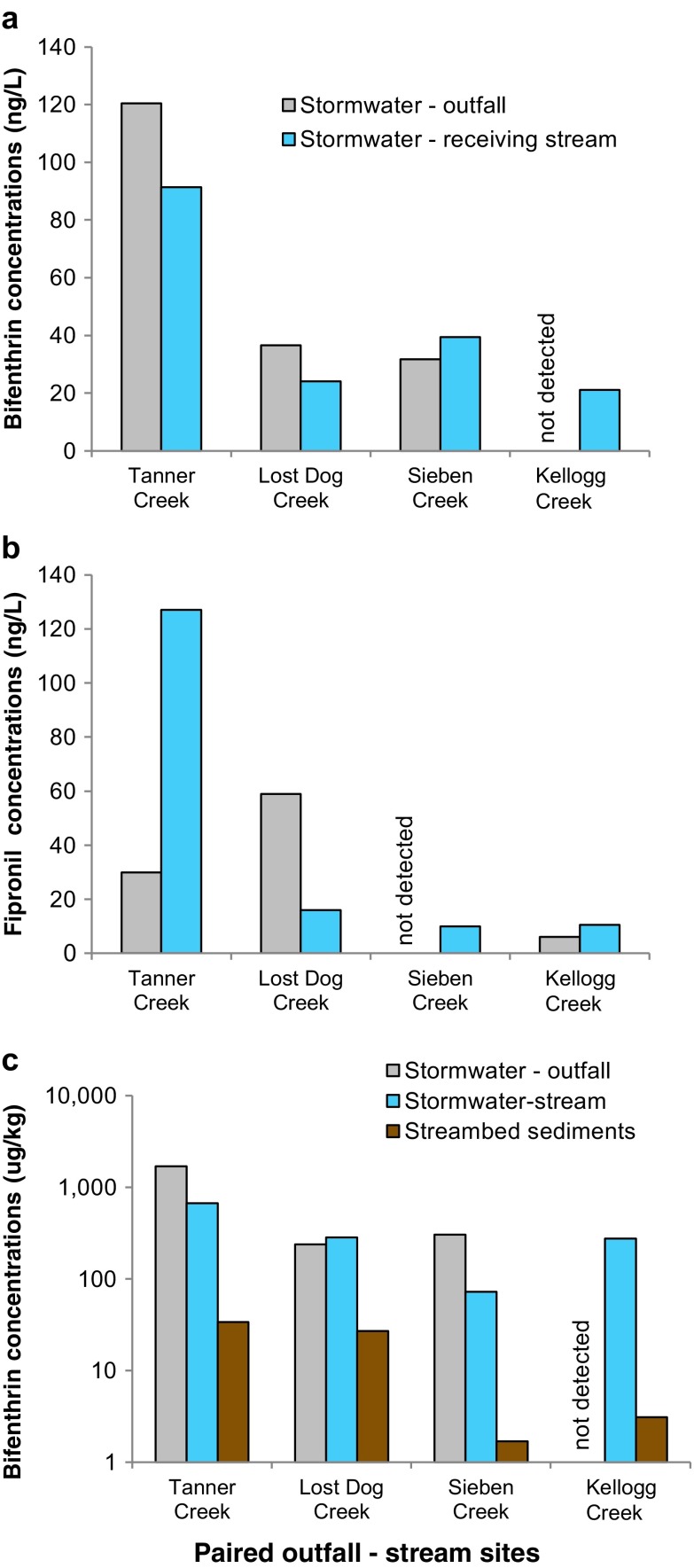
Stormwater runoff concentrations of **a** total bifenthrin and **b** dissolved fipronil in paired stormwater outfalls and receiving streams, and **c** mass-per-unit-mass bifenthrin concentrations in outfalls, streams, and streambed sediments. Note log scale of *y axis* in panel **c**. Lost Dog Creek outfall contains 40 acres of “unpiped” area drained by surface channels

The nested design of the study with four outfall-stream pairs allowed for comparison of pesticide concentrations in outfalls (stormwater runoff and SIFT device sediments) with those in receiving streams (stormwater runoff and streambed sediments) (Fig. 4a–c). In all of the nested pairs, there were some compounds detected in both the outfall and receiving stream, and some compounds detected in one but not the other (SI 13). This could represent compound-specific differences in their upstream sources, timing of pesticide transport relative to sample collection, as well as dilution and fractionation (partitioning to sediment, for example), and, for streambed and SIFT sediments, sediment dilution and (or) degradation.

There were 14 pesticides detected in water in the outfalls, with bifenthrin, fipronil, and metolachlor occurring in over half of samples. In addition, there were 9 pesticides detected in 5 SIFT samples (Table 2). The highest concentrations were for pendimethalin and bifenthrin, which occurred in all 5 SIFT samples; their 100 % detection points to these highly urbanized watersheds as important source areas for these compounds. Most of the pesticides detected in the receiving streams were also discharged by stormwater outfalls. Exceptions included insecticides (cyfluthrin and fenpyroximate) and the fungicide propiconazole, which were detected in one and three streams each, respectively, but not in any outfalls, pointing to other upstream sources.

Thirteen pesticides were detected in streambed sediments, with one to six compounds per stream (Table 3). Bed sediments contained bifenthrin in 71 % of streams, overall, and nearly two thirds contained one or more DDT degradates. With the exception of Tanner and Lost Dog Creeks, these bifenthrin concentrations are similar to those reported by Weston et al. (2011) for streams in the Pacific Northwest, including Kellogg Creek, which was sampled again during our study.

The highest concentrations of bifenthrin occurred in the outfall to Tanner Creek (Table 2), where the concentration more than accounted for that found downstream in the creek (Table 3), pointing to the outfall as an important source. Tanner Creek and the outfall are situated in a neighborhood in close proximity to large residential properties with extensive turf and manicured landscaping that may be treated with bifenthrin and other pesticides. The high concentration in the outfall relative to other sites may reflect recent/fresh applications on upland areas in the neighborhood.

Bifenthrin was also found in Tanner Creek bed sediments (Fig. 4c), at a concentration 20 times lower than that on storm-derived suspended sediment and 50 times lower than the concentration on suspended sediments from the outfall. Taken together, these results indicate the importance of recent inputs of bifenthrin to Tanner Creek from this outfall.

**Fig. 5:**
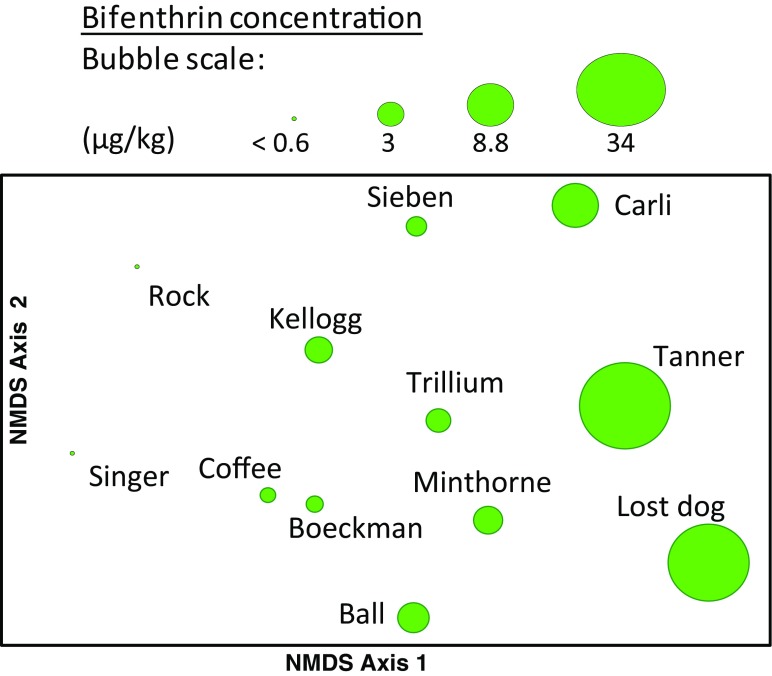
Regression of bifenthrin concentrations on suspended sediments (in runoff) and those in streambed sediments. Note log scale in *x* and *y axes*

The outfall to Lost Dog Creek and downstream site contained similar concentrations of mostly sediment-associated bifenthrin (Fig. 5a), which similarly originates from the outfall’s upstream watershed mostly comprised of residential properties, a golf course, and other possible areas where bifenthrin and other insecticides may be applied. Dissolved concentrations of fipronil and carbaryl were about 3.5 times higher in the outfall compared with the downstream site. The timing of runoff relative to sample collection in this steep watershed may have contributed to such differences in concentrations between the outfall and stream site.

The bifenthrin concentration in bed sediments in Lost Dog Creek was an order of magnitude lower than that found in runoff in both the stream and outfall (Fig. 4c), indicating, once again, fresh inputs as the primary source. In similar fashion, DDE transported on suspended sediments was six times more concentrated than that found in the creek bed sediments. Other pesticides such as cyfluthrin, dithiopyr, pentachloranisole (PCA), and trifluralin were also found in the bed sediments, but not in the upstream outfall, suggesting other sources for these pesticides.

The outfall to Rose Creek was a source of bifenthrin, pendimethalin, and trifluralin to Sieben Creek (Table 2); three other pesticides—dithiopyr, prodiamine, and PCA—were detected in the outfall but not downstream. This outfall drains a shopping center that is nearly 100 % commercial/retail (Andrew Swanson, Clackamas County Water Environmental Services, written commun., 2013), and pesticides applied to landscaping may be washed onto pavement and carried through storm drains to the outfall, which discharges to surface water about 1 mi upstream from the Sieben Creek sampling site (SI 1). The drainage basin for this outfall is small, however, making up just 0.6 % of Sieben Creek’s basin area, which limits its influence on downstream pesticide concentrations. Sieben Creek drains into the lower Clackamas River upstream from four major drinking water intakes, and was previously identified as an important pesticide source (Carpenter et al. 2008).

Although the whole water concentration of bifenthrin at the outfall was slightly lower compared with Sieben Creek downstream, the bifenthrin concentrations on the suspended sediments were four times greater in the outfall (Fig. 4c), suggesting downstream dilution by sediments having, on average, lower bifenthrin concentrations.

Pendimethalin was also detected in the outfall—only in SIFT sediments—at concentrations of 20 μg/kg following the first storm (sample 1) and 849 μg/kg in sample 2 (Table 2). The concentration on suspended sediments in Sieben Creek during the storm was intermediate (46 μg/kg). Three other compounds, dithiopyr, prodiamine, and PCA, were detected in SIFT sediment from the outfall, but not in Sieben Creek downstream, and eight other pesticides, including fipronil, carbaryl, and DDT degradates, were detected in Sieben Creek stormwater, but not in the outfall. These results are not unexpected, as discharge from the outfall makes up only a small fraction of the flow in the creek, and pesticide runoff from residential properties in other parts of the basin are likely occurring.

The outfall to Kellogg Creek was a source of bifenthrin, fipronil, pendimethalin, trifluralin, DDE, and four other pesticides. While bifenthrin was detected in both SIFT sediment samples (Table 2), bifenthrin was below detection in the stormwater runoff sample collected at the outfall. This may have resulted from sampling the outfall after the major flush of sediments had already occurred. The water sample did contain dissolved fipronil (plus a degradate) and metolachlor (Table 3, Fig. 4b), and these moderately-to-highly water-soluble compounds (SI 10) might be expected to linger in the receding stormwater more so than sediment-associated pesticides that settle out when runoff velocities decline. Three of the four pesticides detected in Kellogg Creek bed sediments, bifenthrin, oxyfluorfen, and DDE, were also detected in the stormwater outfall. In addition to the outfall, Minthorne Spring Creek was another source of bifenthrin, fipronil, and metolachlor (Table 3) to downstream Kellogg Creek (SI 1).

### Sources, transport, and fate of bifenthrin, fipronil, and DDT degradates

Bifenthrin, fipronil, and a DDT degradate (DDE) were the insecticide compounds most commonly detected in this study. They represent three chemically distinct classes and have different modes of action upon target organisms, though all have potential to cause adverse effects on aquatic invertebrates in streams.

#### Bifenthrin

Bifenthrin was the most frequently detected pesticide in our study, transported primarily sorbed to suspended sediments (Fig. 3). Almost all (97 %) of the bifenthrin mass transported during the storm was associated with suspended sediments. Bifenthrin was detected in all five outfalls in stormwater or SIFT sediments and in the bed sediments of > 90 % of streams sampled.

Bifenthrin concentrations in streambed sediments were on average 50 times (up to 270 times) lower than those in stormwater runoff, though concentrations were positively correlated (*r*^2^ = 0.71, *p* < 0.001; Fig. 5). While resuspension of sediment-bound bifenthrin in the streambed may occur during storms, the higher concentrations in four out of five outfalls suggest that inputs of fresh chemical from the landscape to receiving streams enriches streambed sediments, but that degradation and (or) sediment dilution result in lower concentrations.

Bifenthrin and DDE were the only compounds detected in both suspended and streambed sediments, a finding that is likely due to their high organic carbon partitioning coefficient (Koc) values and relatively long half-lives (Weston et al. 2011; Saran and Kamble 2008; Gan et al. 2005b; SI 10). Although no data are available to assess the local use of bifenthrin, high non-agricultural use of bifenthrin-containing products was shown for the Puget Sound counties (Washington State Department of Agriculture 2014). Bifenthrin is widely used for control of structural pests—carpenter ants and termites—but it is also approved to control insect pests on residential lawns, golf course turf, and as a broad-spectrum insecticide for landscape ornamentals.

#### Fipronil

Fipronil, a phenyl pyrazole insecticide, was detected in about two thirds of outfalls and streams—all in the dissolved phase—along with a few detections of degradates in outfall samples. Fipronil is often used by professional applicators for structural pests, especially termites and carpenter ants, and for control of larvae and adult cockroaches, mosquitos, locust, ticks, and fleas. Because it has a unique mode of action, fipronil is considered effective for pests that may have become resistant to other insecticides such as pyrethroids, organophosphates, or carbamates (Bobe et al. 1997).

During 2008, fipronil was the most common insecticide applied in Oregon, making up 35 % of the total reported use statewide (Oregon Department of Agriculture 2008). Its frequent detection in our study suggests that fipronil use continues to be important in northwestern Oregon. Fipronil is used exclusively in urban areas and is not applied to agricultural crops (Gunasekara and Troung 2007). Fipronil is moderately soluble and has a relatively low Koc; none was detected on sediments. But, like bifenthrin, fipronil has a relatively long half-life, which, along with its common use, contributes to its frequent detection in urban stormwater.

#### DDT degradates

DDT degradates (DDD and DDE) were commonly detected in these streams, almost entirely associated with sediments—from outfalls, streams, and streambeds (Table 1, Table 2, and Table 3). Though banned in 1972, DDT degradates—toxic, hydrophobic, and bioaccumulative—continue to be detected; in this study, they were found in two thirds of streams, revealing their persistence across much of the study area. For streams where DDE was detected in both the suspended and streambed sediments, concentrations were 5–25 times lower in streambed sediment, suggesting mobilization of higher-concentration sediments from upland sources or possibly bank erosion during high discharge.

Detections of DDE in the Kellogg Creek watershed, including the outfall to Kellogg Creek (Table 2), Minthorne Spring, and Kellogg Creek bed sediments (Table 3), suggest continued transport of these compounds on sediments eroded from the watershed with subsequent deposition in the creeks. Their slow degradation provides opportunity for long-term exposure that may affect stream life.

### Pesticides exceeding benchmarks for invertebrates and water quality criteria

Nearly all stormwater runoff samples (14 of 15 streams) contained one or two insecticide(s) at levels exceeding U.S. EPA OPP chronic benchmarks for invertebrates (Table 1). Concentrations of fipronil and malathion in Tanner and Singer Creeks exceeded U.S. EPA acute benchmarks for invertebrates with respective BQ values of 1.15 and 1.5. While these one-time samples may or may not have characterized peak concentrations, exceedances of acute benchmarks suggest that levels were sufficiently high in these streams to impair invertebrates, at least for a period of time.

Many more insecticide detections potentially exceeded U.S. EPA chronic benchmarks for invertebrates, with bifenthrin and fipronil exceedances in 80 and 46 % of streams, respectively (Table 1). These EPA chronic benchmarks are based on 21-day average water concentrations, not instantaneous concentrations during peak stormwater runoff as reported here. Thus, comparisons to these chronic benchmarks may overestimate actual toxicity to aquatic life if exposures are shorter lived.

Some of these exceedances were, however, well above chronic benchmarks, and likely exceeded chronic values for some time after the storm, depending on the sources, transport, and flushing rates, among other factors. In addition, our screening process used standard single-compound benchmark quotient (BQ) values as potential indicators of invertebrate toxicity, and did not consider possible cumulative effects of mixtures. But, in reality, stream biota are exposed to multiple pesticides (Carpenter et al. 2008) and other pollutants in stormwater including metals and poly-aromatic hydrocarbons (McIntyre et al. 2014), which may collectively produce toxicity for aquatic life. These perspectives support the use of lower, chronic benchmarks as a potentially useful, albeit conservative, screening approach. Taking this approach, there were two to three insecticides (bifenthrin, fipronil, and (or) a DDT degradate) in 40 % of urban streams exceeding chronic benchmarks or water-quality criteria (Table 1).

### Potential effects of bifenthrin on invertebrates

In addition to the potential exceedances of aquatic-life benchmarks in stormwater, other lines of evidence suggest that bifenthrin in particular may be altering invertebrate assemblages in these urban streams. The Bio-ENV BEST multivariate analyses identified bifenthrin concentrations in streambed sediments as the most important variable in the solution (rho = 0.59; *p* < 0.001), explaining a significant amount of variation in the benthic invertebrate similarity matrix. Sites with relatively high concentrations of bifenthrin in bed sediments were dominated by tolerant organisms including amphipods, flatworms, oligochaetes, blackflies, and midges. All of them had severely disturbed invertebrate assemblages (Table 1). Although stormwater concentrations of bifenthrin were higher in runoff compared with bed sediments, runoff concentrations were not significant in the BEST analysis (*p* > 0.05).

**Fig. 6:**
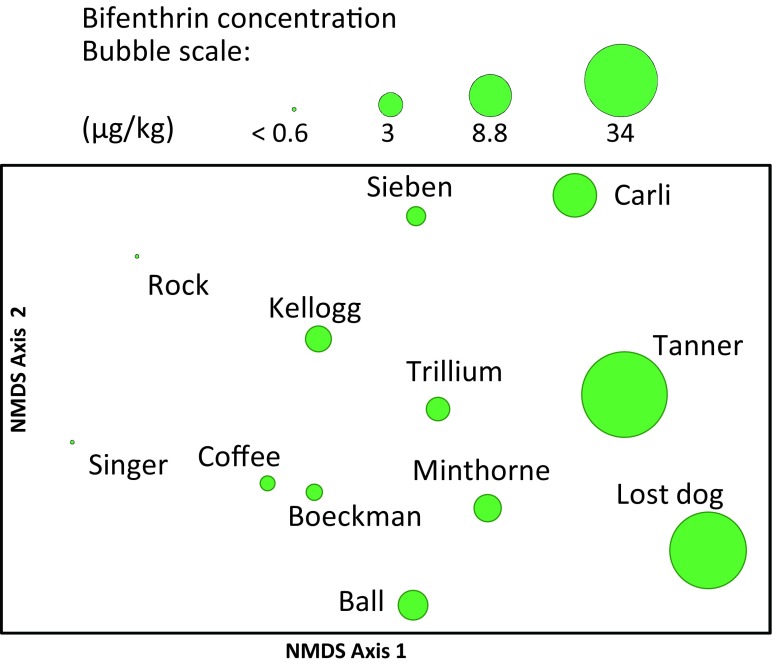
Ordination of creek benthic invertebrate samples with bubble plot overlay of bifenthrin concentrations in streambed sediments

Consistent with these BEST results, bifenthrin in streambed sediments was significantly correlated with NMDS axis 1 (rho = 0.75, *p* < 0.01, Fig. 6). NMDS Axis 1 scores were also significantly correlated (rho = –0.82, *p* < 0.001) with % sensitive EPT (mayflies [but not including those in the *Baetis tricaudatus* complex], stoneflies, and caddisflies). NMDS Axis 2 was significantly correlated (rho = 0.63–0.69, *p* < 0.05) with total invertebrate abundance (insect and non-insects), reflecting the high densities of tolerant organisms at sites such as Carli Creek, with low total abundances overall in Lost Dog and Ball Creeks (Fig. 6, also see Lemke et al. 2013; Cole 2014).

**Fig. 7:**
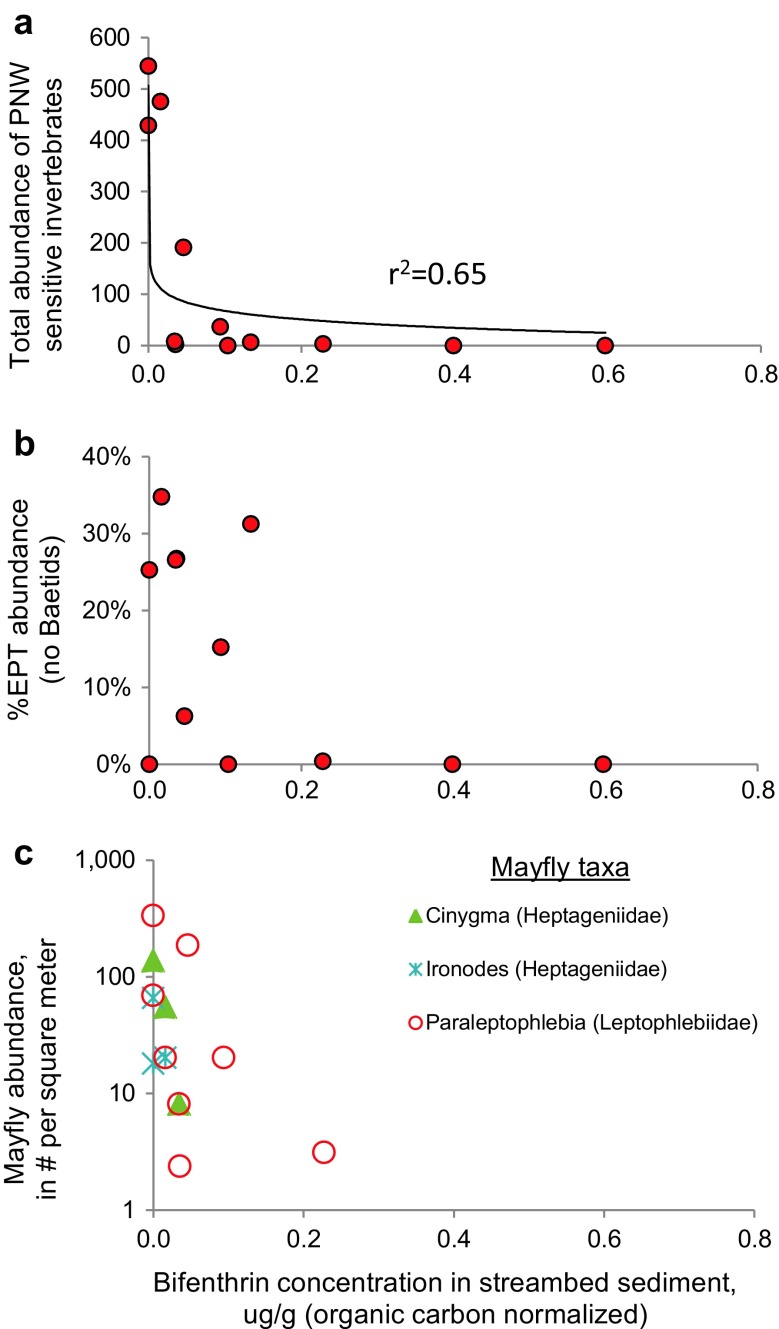
Biplots of organic-carbon-normalized bifenthrin concentrations in streambed sediments and **a** abundance of sensitive invertebrate taxa, **b** percent EPT abundance (see text), and **c** abundance of select mayfly taxa. Includes only streams with comparable invertebrate data. Pacific Northwest [PNW] sensitive invertebrate scores based on tolerance values from Barbour et al. (1999)

In addition, a strong negative response in the total abundance of sensitive benthic invertebrates occurred with increasing bifenthrin (organic-carbon-normalized concentrations) in streambed sediments (Fig. 7a), based on U.S. EPA tolerance values for the Pacific Northwest Region (Barbour et al. 1999). A similar decline in % EPT abundance (not including *Baetis*) and declines in three mayfly taxa were observed with higher bifenthrin concentrations in bed sediments (Fig. 7b, c). *Baetis* (swimming mayflies), including those in the *B. tricaudatus* complex, are often found in disturbed urban streams (Waite et al. 2008). They were removed from the % EPT metric because, unlike most EPT, which are relatively sensitive to environmental conditions, *Baetis* mayflies are more tolerant (Barbour et al. 1999). High abundances of *B. tricaudatus* (complex) can develop due to their relatively short generation time (∼30 days), and because they are common in the drift, they are effective colonizers and may occur in high abundances despite poor water quality or sediment contaminants.

The highest bifenthrin concentration in bed sediments (34 μg/kg) occurred in Tanner Creek, where the invertebrate assemblage disturbance class was rated “severe” (Table 1, also see Cole 2014), with dominance by fast colonizers (*B. tricaudatus* complex) or tolerant non-insect taxa, including flatworms, amphipods, and oligochaete worms. Lost Dog Creek, which had the second highest bifenthrin concentrations in bed sediments (27 μg/kg), had low abundance of invertebrates and an exclusively tolerant assemblage dominated by amphipods, flat worms, oligochaete worms, and midges. Carli Creek had much higher densities of invertebrates despite the third highest bifenthrin concentration (8.8 μg/kg), but was also dominated by tolerant organisms—isopods, black flies, oligochaetes, and *B. tricaudatus* complex.

The negative relationship between bifenthrin concentrations in streambed sediments with indicators of healthy invertebrate populations suggests that bifenthrin could be causing community declines and shifts, but other insecticides such as fipronil, DDT degradates, and other pyrethroids may also contribute to degrading invertebrate populations in these streams (Table 1). In addition, fine sediment and warm water temperatures (Lemke et al. 2013; Cole 2014), or other pollutants, including copper, zinc, and lead, commonly found in urban streams at levels exceeding water quality criteria (Hobbs et al. 2015), also may contribute to the cumulative degradation in these streams.

Bifenthrin is highly toxic to aquatic invertebrates, affecting the central and peripheral nervous system by delaying the closure of the sodium ion channels leading to paralysis and death (Johnson et al. 2010). In addition, sub-lethal toxic effects of pyrethroids, such as reduced growth, altered behavior and endocrine/reproductive effects have also been documented that could affect survival, growth, or reproduction of benthic organisms (Werner and Moran 2008).

A national study of urban streams (Nowell et al. 2013; Moran et al. 2012) examined 108 contaminants in bed sediment and found bifenthrin to be the best single predictor of toxicity to the benthic invertebrates. A recent study by Weston et al. (2014) found that water-column bifenthrin concentrations in the 10–20 ng/L range were enough to impair normal movements in about one third of the dozen invertebrate taxa tested. In many studies, the predicted toxicity of bifenthrin in streambed sediments alone was enough to explain a large proportion of the observed toxicity in experimental tests using amphipods (Weston et al. 2005, 2009, 2011; Amweg et al. 2006; Hintzen et al. 2009). A later study found evidence that amphipod populations in some Californian streams have developed resistance and become desensitized to pyrethroids (Weston et al. 2013), which may explain prevalence of these organisms in some of the more impacted streams, including in this study.

Pyrethroids such as bifenthrin are also more toxic at lower water temperatures (Weston et al. 2011; Holmes et al. 2008). The relatively warm water temperatures that often plague urban streams may, ironically, reduce toxicity of pyrethroids to stream invertebrates; this presents challenges for water and land managers working to re-establish riparian vegetation and shading with the goal of reducing water temperatures.

The occurrence of high numbers of tolerant crustaceans, including isopods, amphipods, and decapods, in streams where bifenthrin and other persistent pesticides occur also raises the potential for these organisms, while tolerating moderate to high concentrations of pesticides in urban streams, to bio-accumulate and (or) transfer these contaminants into the food web. Bifenthrin has been found in tissue samples from amphibians (Smalling et al. 2013a, 2015), fish (Smalling et al. 2013b), crab embryos (Smalling et al. 2010), and brown trout (Bonwick et al. 1996) in other watersheds, but studies are needed to evaluate whether sensitive life stages of endangered salmonids are being affected. Recent research by Weston et al. (2014) in the American River, CA, found that while typical concentrations of bifenthrin and other pyrethroids were not directly toxic to steelhead, rainbow trout, or Chinook salmon, their invertebrate prey were affected, and they concluded that food-web effects are of greatest concern for these fish populations. More study is therefore needed to fully understand the effects of pyrethroids and other pesticides on aquatic life in these streams.

### Potential effects of fipronil on invertebrates

Fipronil was also frequently detected in stormwater runoff, exceeding its chronic benchmark for invertebrates in nearly half of the creeks sampled. Repeated exposures to fipronil may also contribute to degraded invertebrate assemblages in some of these streams. Fipronil is a broad-spectrum insecticide that blocks gamma-aminobutyric acid (GABA)-gated chloride channels in the central nervous system of invertebrates, eventually causing paralysis and death (Jackson et al. 2009). Weston and Lydy (2014) proposed that the largest threat from fipronil to aquatic invertebrates is not solely by causing death directly but also by affecting their movement, swimming, and clinging behaviors, which are important for survival and reproduction.

Two fipronil degradates, fipronil sulfide and fipronil desulfinyl, were also detected—but only in outfall samples (Table 2). Fipronil degradates, especially fipronil sulfide, can be more toxic to aquatic invertebrates than the parent compound (U.S. EPA 1996; Weston and Lydy 2014), and while there are no existing aquatic-life benchmarks for these degradates, their presence and toxicity suggest that it would be worthwhile to include these degradates in future monitoring studies.

### Basin characteristics as predictors of pesticide occurrence

Although none of the pesticide variables correlated with total percent urban or percent impervious area, the highest pesticide concentrations and (or) largest numbers of compounds detected occurred in Tanner and Lost Dog Creeks and their upstream outfalls, and in Sieben, Singer, and Carli Creeks, which all drain highly developed basins (52–96 % urban, Table 1). Tanner and Lost Dog Creek watersheds are generally steeper and have relatively high drainage densities—conditions that produce rapid runoff that transports pesticides to streams.

Housing density was not significantly correlated with any of the pesticide variables except total fungicide concentrations, which were positively correlated with high-density development (*p* < 0.001) and negatively correlated with low-density development (*p* < 0.05). Considering just the urban streams, bifenthrin, fipronil, and carbaryl concentrations in stormwater runoff were positively correlated (*p* < 0.05) with the percentage of developed open space, defined as “vegetation planted in developed settings for recreation, erosion control, or aesthetic purposes” (Fry et al. 2011). This category also includes lawns associated with large-lot single-family houses, parks, golf courses, and cemeteries; impervious areas represent less than 20 %. This suggests that applications to grass turf and (or) landscaping in developed open spaces may be important sources of these insecticides.

## Conclusions

This study was the first to examine a broad range of pyrethroid insecticides and other current-use pesticides in stormwater runoff and streambed sediments in urban streams in northwest Oregon. Numerous pesticides were detected in stormwater runoff and (or) streambed sediments, with two insecticides—fipronil and malathion—occurring at concentrations exceeding EPA acute benchmarks for aquatic invertebrates. Concentrations of bifenthrin exceeded the EPA 21-day chronic benchmark for invertebrates, though reported concentrations were instantaneous values that may overestimate potential toxicity if these elevated concentrations were short lived. Comparing bifenthrin and other insecticides to chronic benchmark values is conservative because benchmarks do not take into account the typical pattern of exposure to multiple pesticides. Applying these chronic benchmarks, 40 % of stream potentially exceeded two aquatic-life benchmarks or the DDT-plus-degradate water quality criterion simultaneously. The potential effects of DDT degradates on aquatic life have not been investigated in detail in this part of Oregon, but their frequent occurrence in stormwater and streambed sediments, combined with their relatively high toxicity, suggests that additional monitoring may be warranted in some of these basins.

Comparisons of pesticide occurrence and concentrations between outfalls and streams provided by the paired sampling design begin to shed light on the contributions from stormwater outfalls to streams during runoff periods, although only one storm event was sampled, and at a limited number of sites. Higher concentrations in the outfalls compared with streams suggest dilution downstream, whereas higher concentrations in streams compared with their paired outfall suggest additional sources upstream that were not sampled, including other outfalls and nonpoint sources.

Streams in the cities of Lake Oswego and West Linn generally had the highest concentrations of bifenthrin and fipronil. This is likely attributable to rapid transport of pesticide-laden runoff from application areas, a process facilitated by relatively steep slopes, high amounts of impervious surfaces, and relatively high drainage density. This, combined with the relatively long half-lives, allows these pesticides to reach streams prior to degradation.

The poor quality of the invertebrate assemblages in the MS4 streams in 2011 and 2013 (Lemke et al. 2013; Cole 2014) indicates a substantial degree of impairment, consistent with multiple stressors that likely include pesticides. Bifenthrin concentrations in streambed sediments were negatively correlated with several benthic invertebrate metrics that suggest impairment to both abundance and composition of sensitive types of invertebrates (e.g., EPT taxa) that are important prey for salmonids and other fish, birds, bats, and other animals. While the statistically significant correlations presented herein do not prove or demonstrate cause and effect, especially considering the small number of samples, they suggest that bifenthrin may have substantial effects. While the limited duration and scope of our study preclude reaching unequivocal conclusions about the effect of bifenthrin on invertebrates in these streams, our results contribute to a growing body of scientific research linking pyrethroid insecticides—bifenthrin in particular (Moran et al. 2012; Weston et al. 2014)—to toxic effects on stream invertebrates.

Given the strong tendency for pyrethroids to sorb strongly to sediments, analysis of dissolved compounds alone will not be effective at detecting these current-use pesticides except at very high concentrations. The SIFT devices were effective for sampling sediments in the outfalls, producing a 100 % detection rate for bifenthrin. Future monitoring of sediment-bound bifenthrin could examine sources of these hydrophobic insecticides in more detail; such knowledge could enhance existing stormwater management infrastructure and inform future development of Best Management Practices (BMPs) aimed at reducing pesticide occurrence in these and other urban streams in the USA and across the globe.

Because of the temperature dependence on the toxicity of pyrethroid insecticides—with greater toxicity at lower temperatures (Holmes et al. 2008; Weston et al. 2011)—it is important that laboratory studies evaluating toxicity to aquatic life be conducted at ambient stream temperatures to obtain real-world results. The organic carbon content and (or) the mineral/biological character of sediments (Weston et al. 2011) may also play an important role in the transport, bioavailability, and toxicity of bifenthrin and other pesticides to invertebrates. Deciphering these details could lead to a better understanding of how and where pyrethroids are most likely to be transported in urban watersheds, and the effects they may have on stream invertebrates at ambient temperatures.

Stormwater management is an ongoing endeavor in temperate urban areas. Despite strategies and regulations to reduce pollution during stormwater runoff, sediment and pollutants including pesticides continue to enter streams where they appear to have substantial effects on benthic invertebrates. Because these organisms are an important food resource for endangered fish and other wildlife, it is critical to better determine the full impact from these current-use insecticides on aquatic life so that mitigation solutions can be implemented.

## Electronic supplementary material

Below is the link to the electronic supplementary material.

ESM 1 Map of study area showing locations of streams and stormwater outfalls. (ODS 221 kb)

ESM 2 List of stormwater outfall and stream sampling sites, basin land use, and location. (ODS 17 kb)

ESM 3 Continuous streamflow and turbidity conditions in nearby Fanno Creek. Arrow indicates storm sampling in the urban creeks. (ODS 50 kb)

ESM 4 Laboratory analytical method detection limits for sediment and water. (ODS 20 kb)

ESM 5 Quality-assurance results for field and lab blank samples for dissolved and suspended sediment fractions in stormwater and bed sediment. (ODS 20 kb)

ESM 6 Quality-assurance results for three replicate sample pairs. (ODS 19 kb)

ESM 7 Quality-assurance results showing percent recoveries for matrix-spiked samples. (ODS 25 kb)

ESM 8 Percent recoveries for surrogate spike samples for dissolved and suspended sediment associated pesticides. (ODS 20 kb)

ESM 9 Potential uses of pesticides detected and example products. (ODS 18 kb)

ESM 10 Properties of pesticides detected. (ODS 21 kb)

ESM 11 Dissolved pesticide concentrations in stormwater runoff samples, September 2013. (ODS 22 kb)

ESM 12 Suspended-sediment associated pesticide concentrations in stormwater runoff samples. (ODS 21 kb)

ESM 13 Comparison of pesticide detections in outfalls and receiving streams. (ODS 20 kb)
